# Bayesian Spatial Survival Models for Hospitalisation of Dengue: A Case Study of Wahidin Hospital in Makassar, Indonesia

**DOI:** 10.3390/ijerph17030878

**Published:** 2020-01-30

**Authors:** Aswi Aswi, Susanna Cramb, Earl Duncan, Wenbiao Hu, Gentry White, Kerrie Mengersen

**Affiliations:** 1 ARC Centre of Excellence for Mathematical and Statistical Frontiers, Queensland University of Technology, 2 George St, Brisbane, Queensland 4001, Australia; susanna.cramb@qut.edu.au (S.C.); earl.duncan@qut.edu.au (E.D.); gentry.white@qut.edu.au (G.W.); k.mengersen@qut.edu.au (K.M.); 2 School of Public Health and Social Work, Institute of Health and Biomedical Innovation, Queensland University of Technology, Victoria Park Road, Kelvin Grove, Queensland 4059, Australia; w2.hu@qut.edu.au

**Keywords:** dengue fever, Bayesian spatial survival model, Weibull model, Cox model, Leroux model, conditional autoregressive prior

## Abstract

Spatial models are becoming more popular in time-to-event data analysis. Commonly, the intrinsic conditional autoregressive prior is placed on an area level frailty term to allow for correlation between areas. We considered a range of Bayesian Weibull and Cox semiparametric spatial models to describe a dataset on hospitalisation of dengue. This paper aimed to extend these two models, to evaluate the suitability of these models for estimation and prediction of the length of stay, compare different spatial priors, and determine factors that significantly affect the duration of hospital stay for dengue fever patients in the case study location, namely Wahidin hospital in Makassar, Indonesia. We compared two different models with three different spatial priors with respect to goodness of fit and generalisability. For all models considered, the Leroux prior was preferred over the intrinsic conditional autoregressive and independent priors, but Cox and Weibull versions had similar predictive performance, model fit, and results. Age and platelet count were negatively associated with the length of stay, while red blood cell count was positively associated with the length of stay of dengue patients at this hospital. Using appropriate Bayesian spatial survival models enables identification of factors that substantively affect the length of stay.

## 1. Introduction

Time-to-event or survival analysis is a set of statistical procedures for analysing data for which the outcome variable is time until an event occurs. In biostatistics, the event is often death, recovery or disease incidence, and survival time is usually defined in days, weeks, or years. A key feature is that not all individuals will experience the event of interest within the given period of the study, so some cases are censored [1,2].

Data used in survival analyses are often collected over distinct spatial regions. If there is the potential for survival time to vary between locations, it may be useful to include spatial information in the survival model. Bayesian spatial survival models have recently emerged in the literature, and commonly spatially structured priors are placed on area-specific frailty terms [3]. An early example is the study of breast cancer and malignant melanoma patients using a fully Bayesian Cox model by incorporating spatial autocorrelation between neighbouring areas [4]. A Bayesian spatial survival model that included conditional autoregressive (CAR) distributed area-specific random effects (frailty) using a parametric proportional hazards model with a Weibull distribution for the baseline hazard was developed for infant mortality data [5].

Here, we follow and extend the Bayesian approaches of Banerjee et al. [5] for a Weibull survival model and Osnes and Aalen [4] for a semiparametric Cox proportional hazards model to describe hospitalisation for dengue fever in a hospital in Makassar, Indonesia. This case study is described in detail below. Although spatial patterns in dengue fever have been the subject of considerable research effort [6], there appears to have been little research so far into modelling time-to-discharge for dengue hospitalisations. Hospitalisation of dengue fever patients is expensive [7] and understanding the geographic pattern of hospitalisation, duration of hospitalisation, influential factors affecting hospitalisation, and the influence of the severity of presentation at the hospital, is critical for hospital management. This information can also be used by the health manager and researchers to help to understand the dynamics of dengue and guide targeted interventions to reduce the disease. 

The clinical course of dengue is classified into three different categories: A febrile phase (0–3 days), a critical phase (4–6 days), and a recovery phase (7–10 days) [8]. The incubation period within mosquito ranges from 8 to 12 days, and within humans from 4 to 10 days [9]. 

In a recent study, factors related to the length of stay (LOS) in the hospital for dengue patients in Sri Lanka using a proportional hazards survival model was analysed by Karunarathna and Sooriyarachchi [10]. These factors were age, sex, ethnicity, place treated initially (private, government hospital, etc.), white blood cell count, platelet count, dengue classification, packed cell count, and district of patient residence. They found that factors associated with hospital LOS for dengue patients were age, dengue classification, ethnicity, platelet count, and districts. However, there was no consideration of the inclusion of spatial random effect in their model nor Bayesian methods. 

A number of studies have used Bayesian survival models to investigate the hospitalisation for dengue in Indonesia [11,12,13,14]. Two studies used a Weibull spatial survival model with a CAR frailty introduced by Banerjee et al. [11,14]. However, these papers implemented only one form of spatial prior (a CAR prior) and focused only on important factors associated with dengue. Other authors used a Bayesian Weibull survival model for dengue patients in Makassar, Indonesia, but this study did not consider including a spatial term in their model [12,13]. None of these papers appeared to examine how well the predictive values from the model fit the observed data which is critical for estimation and prediction. 

The overall objective of this study is to expand on the research described above in order to better describe spatial time to event for hospitalisations of dengue, with a focus on understanding dengue fever in Makassar, Indonesia. This research has three aims: Evaluate the suitability of these models for estimation and prediction of LOS, compare different spatial priors, and determine factors that significantly affect the duration of hospital stay for dengue fever patients. We focus on one hospital, namely Dr Wahidin Sudirohusodo hospital, which has the most complete data, with the intention of including other hospitals later.

## 2. Materials and Methods

### 2.1. Data Collection

Data on admitted dengue cases were obtained from patient medical records from a major public hospital in Makassar, namely Dr Wahidin Sudirohusodo, following ethics approval from the Queensland University of Technology (QUT) University Human Research Ethics committee (number: 1800000560) and written permission from the hospital. The data include inpatient dengue fever and dengue haemorrhagic fever (DHF) cases admitted during 1 January 2013 to 31 July 2018.

The information collected was the LOS (*t*) for dengue fever patients in days, the patient’s condition when discharged from the hospital (recovered/improved, not recovered, died, transferred to another hospital), sex, age, white blood cell (WBC) count, red blood cell (RBC) count, levels of haemoglobin (HGB), haematocrit (HCT), platelet (PLT) count, and address of patient residence. Haematocrit is defined as the proportion of red blood cells to the total blood volume. Residential addresses at admission were geocoded and assigned to a district, which was one of 14 districts in Makassar city in 2017. Wahidin hospital is one of the main referral hospitals in Eastern Indonesia. As a result, many patients come from outside Makassar city. As our spatial analysis was focused on Makassar, only residents of Makassar city were included in the study. Furthermore, hospitalisation was defined as stay in hospital for at least one day, so outpatients (those who visited the hospital but did not stay overnight) were not included. At time zero, survival is 100%; because time is in discrete days, we cannot have patients enter and leave the study at time 0.

The initial dengue hospitalisation from Wahidin hospital comprised 2500 patients. Of these, 1603 (64%) patients came from outside Makassar city and 20 (0.8 %) patients had a LOS of zero days. There were 171 (6.8 %) dengue cases without clinical variables. There was one outlier, a patient who stayed 48 nights, that was also excluded. As a result, the final dengue fever patient dataset used in the analysis included 705 patients. All covariates were standardised to have zero mean and standard deviation of unity to equalise the data variability and range.

The response variable is the LOS for inpatients with dengue fever who enter during the study period, with the following conditions.

1. If an inpatient is released from the hospital because they have recovered and this discharge is within the study period, their survival time (i.e., LOS) is categorised as uncensored survival data since they experienced the event of interest—a successful discharge from the hospital.
2. If an inpatient instead experiences one of the following: Death, transfer to another hospital, exceeds the study end date (31 July 2018), or they self-discharge, then the hospital LOS is censored. As the admission date was always within our study time period, this means that only right censoring is present.

Before undertaking the analysis, the dataset was cleaned, and the accuracy of the data was checked. Any issues found were confirmed with the medical record officer.

### 2.2. Model Formulation

We investigate both parametric models using the Weibull distribution to describe the response variable and semiparametric models using Cox proportional hazards (PH) approach due to their popularity in time-to-event modelling. Unlike the parametric model, the semiparametric approach does not assume any particular form for the baseline hazard. 

Frequentist versions of both Weibull and Cox models were initially run in R version 3.6.1 (The R Foundation for Statistical Computing, Vienna, Austria) [15]. We checked the PH assumption for each covariate using statistical tests based on the scaled Schoenfeld residuals by using a survival package, cox.zph function in R. Age, sex, and platelet count met the PH assumption (Supplementary Material Table S1).

#### 2.2.1. Bayesian Weibull Model

We consider a fully parametric model with proportional hazards. The distribution of the LOS (survival time) for dengue patients was assumed to follow a 2 parameter Weibull distribution. The probability density function of this distribution is given by:

$$f\left(t|b,\;\lambda \right)=\lambda bt^{b-1}e^{-\lambda t^{b}},\;t>0,\;\lambda >0,\;\mathrm{and}\;b>0$$

with a mean equal to $E\left(t\right)=\lambda^{-\frac{1}{b}}\Gamma \left(1+\frac{1}{b}\right)$, where Γ represents the gamma function, *b* is a shape parameter, and λ is a positive scale parameter. The shape parameter *b* indicates a monotonic hazard in the Weibull: *b* >1 indicates that the hazard rate will be increasing monotonically with time; and conversely, *b* < 1 indicates that the hazard rate will be decreasing monotonically with time; and *b* = 1 indicates a constant hazard rate or constant value λ and in this case, the hazard rate is an exponential distribution [16].

In many cases, time to event can be collated into strata based on specified factors such as clinical sites, clinical stages of the disease, characteristics of the patients etc. [5]. Let $t_{k}$ be the time until recovery for individual k, k=1, 2, 3, …K. Let $x_{k}$ be a corresponding vector of covariates. The hazard rate $h\left(t_{k};x_{k}\right)$ for the Weibull model (commonly referred to as the PH parameterisation) is a function of a baseline hazard $h_{0}\left(t_{k}\right)$ and covariates [5,16].

$$h\left(t_{k};x_{k}\right)=h_{0}\left(t_{k}\right)\exp\left(\beta^{T}x_{k}\right),$$

where $h_{0}\left(t_{k}\right)$ = $bt_{k}^{b-1}$, and β contains an intercept term. 

The inclusion of a random effect or frailty term is one strategy to account for possible unobserved heterogeneity. If there are unobserved or unmeasured frailties among observations in the sample, then the hazard rate in the frailty model will be a function of both covariates and the random effect $u_{{\mathrm{area}}_{k}}$ where ${\mathrm{area}}_{k}$ takes a value in *i* = 1, 2, … *I* associated with each individual *k* [16].

$$h\left(t_{k};x_{k}\right)=bt_{k}^{b-1}\exp\left(\beta^{T}x_{k}+u_{{\mathrm{area}}_{k}}\right),$$

$$t_{k}\sim \mathrm{Weibull}\left(b,\lambda_{k}\right)$$

b~Gamma(2,0.5),

β~N(0,100)

where

$$\lambda_{k}=\exp\left(\beta^{T}x_{k}+u_{{\mathrm{area}}_{k}}\right)\;\mathrm{and}\;u_{{\mathrm{area}}_{k}}\;=\;u_{i}$$

was given different spatial priors (see Section 2.2.3).

#### 2.2.2. Bayesian Cox Model

The semiparametric Cox model [17] is the most common choice for modelling time-to-event data, in which no specific parametric form is specified for the underlying baseline hazard. Here, we follow and extend the approach of Osnes and Aalen [4] for a semiparametric Cox proportional hazards model as follows. The model of the LOS is assumed to follow a proportional hazard function (also called intensity function), for patient *k* = 1, 2, …, *K* with covariate $X_{k}$ [4]:

(1) $$h\left(t_{k};x_{k}\right)=h_{kj}=h_{0}\left(t_{k}\right)\exp\left\{\beta^{T}X_{k}\right\}$$

where $h_{0}\left(t_{k}\right)$ is the unknown baseline hazard rate (nonparametric), β is the vector of regression parameters, and both β and X are assumed to be constant over time $j=t_{k}$.

The analysis of counting process data, for example survival data, is usually based on intensity modelling. For patient *k* = 1, 2, …, *K*, $I_{kj}$ is the intensity function (hazard function), $Y_{kj}$ is the at risk indicator, the time of observation of patient *k* within the *j*-th interval of time, $N\left(t_{k};X_{k}\right)=N_{kj}$ is the number of failures in interval [0, *j*]. $dN_{kj}$ is the increment of $N_{k}$ over the small-time interval $\left[j,j+d_{j}\right]$.

$$dN_{kj}=\{\begin{array}{cc} 1 & ;\;\mathrm{if}\;\mathrm{patient}\;k\;\mathrm{is}\;\mathrm{discharged}\;\mathrm{well}\;\mathrm{in}\;\mathrm{time}\;\mathrm{interval}\;j\\ 0 & ;\;\mathrm{otherwise} \end{array}$$

Where $dN_{kj}$ is assumed to have Poisson distribution with a mean of $I_{kj}=Y_{kj}h_{kj}$

$$dN_{kj}\sim \mathrm{Poisson}\left(I_{kj}\right).$$

Consider the multiplicative intensity model in (1):

(2) $$I_{kj}=Y_{kj}\exp\left\{\beta^{T}X_{k}\right\}h_{0}\left(t_{k}\right)$$

We extend this model (2) by adding the spatial frailty term $u_{i}$ using three different priors, namely ICAR, Leroux, and independent priors.

From Equation (2)

$$I_{kj}=Y_{kj}\exp\left\{\beta^{T}X_{k}+u_{i}\right\}h_{0}\left(t_{k}\right)$$

$$h_{0}\left(t_{k}\right)\;\sim \;\mathrm{Gamma}\left(ch_{0}^{*}\left(t_{k}\right),c\right)$$

c=0.001

β~N(0,100)

$h_{0}^{*}\left(t_{k}\right)$ is as a prior guess of the hazard function, and *c* is the degree of confidence in that guess.

#### 2.2.3. Spatial Prior Formulation

We used three different prior formulations: The Leroux prior [18], intrinsic conditional autoregressive (ICAR) prior [19], and an independent prior (vague normal distribution equating to no spatial structure) for both Weibull and Cox models. The Leroux prior adjusts the strength of local neighbourhood spatial autocorrelation by a constant value ρ, as follows.

$$\left(u_{i}|u_{j},\;i\neq j,\;\tau_{u}^{2}\right)\sim N\left(\frac{\rho \sum_{j}u_{j}\omega_{ij}}{\rho \sum_{j}\omega_{ij}+1-\rho },\;\frac{\sigma_{u}^{2}}{\rho \sum_{j}\omega_{ij}+1-\rho }\right)$$

$$\omega_{ij}\;=\;1\;\mathrm{if}\;i,\;j\;\mathrm{are}\;\mathrm{adjacent},\;\omega_{ij}\;=\;0\;\mathrm{otherwise}.$$

ρ~Unif(0,1)

$$\sigma_{u}^{2}\sim \mathrm{IG}\left(1,0.1\right)$$

When ρ = 1 this prior reduces to the intrinsic CAR and when ρ = 0 it reduces to the independent prior. For the Leroux model, the value of ρ is estimated, while for the intrinsic CAR and independent models, it is held fixed. The independent prior does not assume any dependencies between the areas. It has no specific term to describe any spatial autocorrelation, so the error is simply modelled by a spatial unstructured frailty. However, the Leroux prior has a single frailty term that adjusts the strength of spatial autocorrelation between areas by a constant ρ where the values of ρ are between 0 and 1. The ICAR prior is a specific case when ρ=1. 

#### 2.2.4. Sensitivity Analysis

A sensitivity analysis was undertaken to investigate the influence of the priors on the posterior estimates. We considered four different options for the prior on the variance of the spatial frailty, $\sigma_{u}^{2}$, including the precision (1/sigma^2) for gamma (G) and standard deviation (sigma) for the uniform (U) distributions with different hyperparameter values for both the Weibull–Leroux and Cox–Leroux models: G(1, 0.1), G(0.5, 0.05), G(2,0.2), and U(0,5). These hyperparameters produce priors that range from quite concentrated to quite disperse, with respect to the data and posterior distribution. The results showed that the estimates were insensitive to the choice of priors. Two different normal (N) priors for *β* were also considered, namely, *N*(0,100) and *N*(0,1000) for both the Weibull–Leroux and Cox–Leroux models. The results revealed that the posterior estimates were relatively insensitive to all of the above choices and the substantive inferences resulting from the analyses were unchanged. 

### 2.3. Statistical Analysis

The analysis undertaken in this study started with a descriptive analysis of Wahidin hospital. In order to achieve the first research aim, the models were evaluated using two approaches. The first was to compare predictive fit between models using the Deviance Information Criterion (DIC) [20] and Watanabe–Akaike information criterion (WAIC) [21], where a smaller value of DIC and WAIC is preferred. The second was to compare the model goodness of fit, which is less commonly examined. This was achieved through graphical examination of the observed data and modelled results. 

To address the second aim, three different priors were used, namely the ICAR prior, Leroux prior, and independent prior. This is in contrast to previous studies on this topic which only used the ICAR prior. 

To address the third aim, for the selected model, variables were considered to be important if the 95% posterior credible interval (CI) for the un-exponentiated covariate coefficient does not contain zero (i.e., the corresponding CI for the exponentiated covariate coefficient does not contain one).

### 2.4. Implementation

The model parameters were estimated using the R2WinBUGS package in R version 3.6.1 [15]. Posterior quantities for parameters were based on 5000 MCMC samples collected after a burn-in of 5000 samples. R code is available in Supplementary Materials Code S1 and Code S2. 

In WinBUGS, right censoring can be modelled using the function I() [22]. The survival distribution is thus a truncated Weibull. Patients who are censored are given a missing value, whilst patients who recover (experience successful discharge from hospital) are given a zero in the censoring time vector (see Supplementary Material Code S1). 

## 3. Results

### 3.1. Descriptive Analysis of Dr. Wahidin Sudirohusodo Hospital

**Figure 1:**
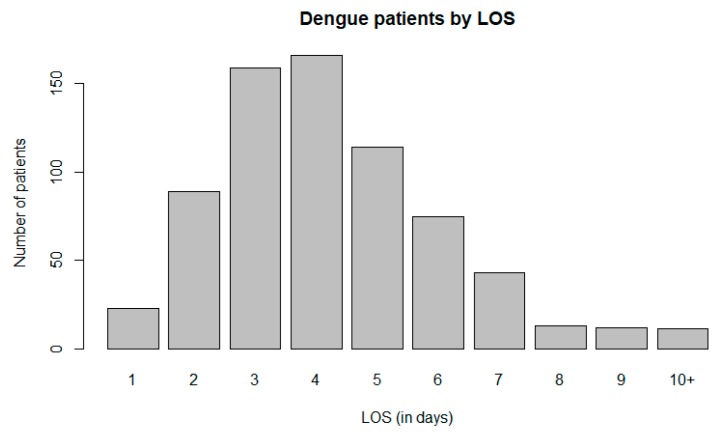
Dengue patients in Wahidin hospital by the length of stay (LOS).

Dr Wahidin Sudirohusodo hospital (Wahidin hospital hereafter) is situated in the Tamalanrea district. The proportion of dengue patients across the 14 subdistricts admitted to this hospital can be seen in Table 1. Most of the patients (approximately 31% of Makassar inpatients) come from the Tamalanrea district followed by the Biringkanaya and Rappocini districts (about 17% each). Fifty five percent of dengue patients are males. The mean LOS is 4.28 days (Table 2), and the most common length of stay is four days (23.5 %), followed by three days (22.5%), and five days (16.1%) (Figure 1).

Most Makassar residents admitted to Wahidin hospital with dengue fever are young, with half being 18 years or less, and 75% being 26 years or less (Table 2). A large variation was observed for clinical variables, with most ranging from abnormally low to abnormally high (Table 2).

### 3.2. Posterior Summaries and Estimation of Spatial Frailty of Weibull and Cox Models

**Figure 2:**
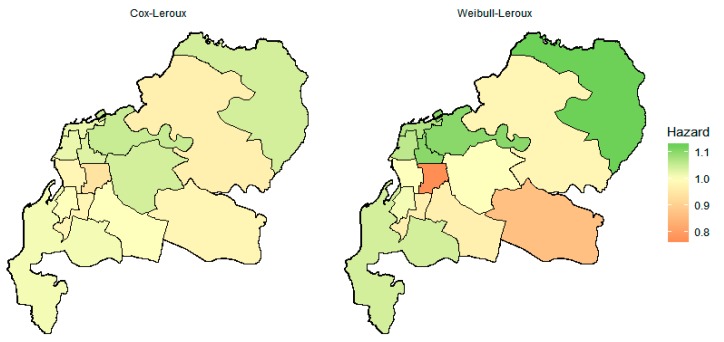
Spatial hazard ratios in each district for Cox and Weibull Bayesian models with the Leroux prior.

The posterior summaries and the spatial random effect (spatial frailty) estimates under the Weibull and Cox models are given in Table 3 and Table 4, respectively. Based on Table 3, it is clearly seen that covariates that were substantively associated with the LOS (that is, 95% credible intervals did not include one) were the same for all three types of priors, that is, age and PLT (negatively associated with the LOS), as well as RBC (positively associated with the LOS). Table 4 shows that all 95% credible intervals of spatial random effects contained one, except for the Weibull model with an ICAR prior, suggesting little variation in LOS between areas after adjusting for these covariates. This indicates that on average, dengue patients from all districts admitted to Wahidin hospital have the same rate of recovery and hospital discharge. This similarity between regions after covariate adjustment is shown in Figure 2.

Overall, from Figure 2, we can conclude that the spatial pattern for Weibull and Cox models are broadly similar, but the Weibull–Leroux model is less smooth compared with the Cox–Leroux model. This is despite both Weibull and Cox having similar estimates for the smoothing parameter ρ=0.62, suggesting similar amounts of spatially structured and unstructured smoothing between them.

Table 5 shows the predictive fit of the Weibull and Cox models based on DIC and WAIC. The Leroux prior was preferred for both the Weibull and Cox models over the other priors considered. Based on DIC and WAIC, Weibull had a better predictive fit over Cox.

**Figure 3:**
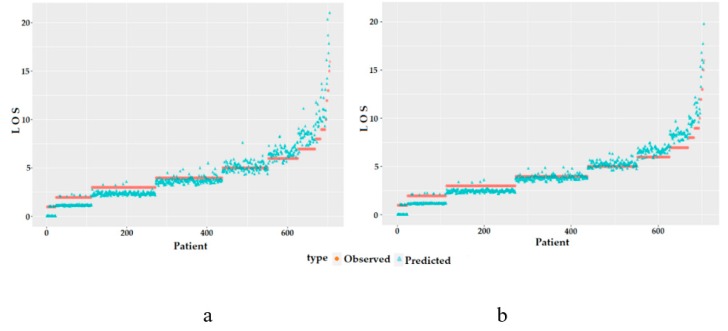
Plots of fitted values versus observed values of the (**a**) Weibull and (**b**) Cox models using a Leroux CAR prior.

A plot of fitted values versus observed values under the Weibull and Cox models using the Leroux CAR prior is presented in Figure 3. Despite differences in model fit, they are visually similar. 

## 4. Discussion

In this study, we have presented an approach to modelling spatial dependence in hospitalisation of dengue. In survival models, it is important not to just consider the relative goodness of fit between models, but it is also important to compare how well the model estimates fit the data. 

Based on the DIC and WAIC, the Weibull model provided a better predictive fit than the Cox model. The results of Tehnizi and Ayatollahi [23] agree with these results. They compared a Cox model and six parametric models to predict time-to-event of leukaemia patients and found that parametric models were better than a Cox model. Khan and Ababneh [24] also found through a simulation study that when the data are applicable for both Weibull and Cox models, the Cox estimates are less accurate than the Weibull estimates. Furthermore, when the Weibull model is applicable to a dataset, then a Cox model could be used for the same dataset as an equivalent model. 

Even though the DIC and WAIC of the Weibull model are less than the corresponding values for the Cox model, the estimates of the parameters are very similar. Both Weibull and Cox models for all three priors indicate that age and platelet count were negatively associated with the LOS, while RBC were positively associated with the LOS for all three types of priors. The negative association between age, PLT, and the LOS means that dengue fever duration and the LOS were prolonged in children. One possible reason is that the immune system in children tends to be more vulnerable than for mature people. Furthermore, the lower the platelet count of dengue patients, the longer the LOS of these patients. The positive association between RBC and the LOS means that dengue patients stay longer in the hospital when their RBC count is high. This is in line with previous studies that have found that patients with a low platelet count with high haematocrit levels are at very high risk of developing severe dengue [25]. Although our models had the association for RBC count, rather than haematocrit, there was high correlation between these two measures, so our results are consistent. 

For all models considered, the Leroux prior was preferred over the ICAR and independent priors. The DIC and WAIC results were similar for the Leroux and independent priors, despite the ρ values indicating that some spatially structured variation was included. This may be because the sample size is small. To our knowledge, no previous dengue survival models have used the Leroux prior to the model spatial frailty in survival. This study demonstrates that including spatial structure may outperform an independent prior even when there is no substantive spatial variation present. The importance of a spatial frailty model is in agreement with some other studies. For example, Darmofal [26] reported a result similar to that found in our study, that the Weibull model with spatial shared frailties (in the form of an ICAR prior) outperforms the Weibull model with no spatial shared frailties. 

The analyses in this paper utilised WinBUGS and R2WinBugs since these packages are well established and supported, and are used by a wide community of analysts. Moreover, the GeoBugs extension in WinBUGS is user-friendly and allows for a variety of spatial prior formulations. The packages OpenBUGS and R2WinBugs could be used in an analogous manner. We deliberately chose these software options over alternatives such as JAGS, as WinBUGS and OpenBUGS provide the ability to implement recursive relationships among the stochastic nodes, which is necessary in spatial models, whereas JAGS explicitly prohibits this kind of model structure. Likewise, Stan has no built-in appropriate spatial prior. This study has several strengths. To our knowledge, this is the first study to use both Bayesian Weibull and Cox models with three different spatial priors for modelling dengue survival. We provide the first report using these models on modelling the LOS of dengue fever cases in a major hospital in Makassar, Indonesia. We include comprehensive clinical information in the model and did not categorise the covariates to prevent information loss. However, our results only used data from one major hospital in Makassar, Indonesia. As a result, some districts have only a few cases. We acknowledge that using other hospitals may affect the results. For example, we may see a strong spatial pattern. Hospitalisation data do not represent all dengue incidence cases, since some cases are not sufficiently severe to warrant hospitalisation. Our future work aims to examine other hospitals as the curated data becomes available. Another limitation of this study is related to missing clinical records, although this only affected 6.8% of cases.

## 5. Conclusions

In summary, the key finding is that Weibull and Cox models provided similar results for this case study. The Weibull model provided better fit in terms of DIC and WAIC, and overall, the Leroux prior was preferred over the intrinsic CAR prior and independent prior. This study also found that covariates that were substantively associated with hospitalisation for dengue (that is, that the corresponding coefficients were substantively different from one, such that the posterior 95% credible intervals did not include one) were the same for the three Leroux, ICAR, and independent priors. Age is an important demographic covariate, while platelet and red blood cell count are important clinical covariates. Although no substantive spatial structures were found, using a Leroux spatial model gave advantages in model performance. Including other hospitals and comparing the duration of the length of stay between hospitals could be potential future work. 

## Figures and Tables

**Table 1:** The distribution of dengue patients admitted to Wahidin hospital in the study period by district of residence.

	Districts	Number of Dengue Patients	Proportion
1	Biringkanaya	117	16.6
2	Bontoala	9	1.3
3	Makassar	19	2.7
4	Mamajang	15	2.1
5	Manggala	73	10.3
6	Mariso	13	1.8
7	Panakkukang	59	8.3
8	Rappocini	117	16.6
9	Tallo	17	2.4
10	Tamalanrea	**219**	**31.1**
11	Tamalate	28	3.9
12	UjungPandang	12	1.7
13	UjungTanah	<5	<0.7
14	Wajo	<5	<0.7
Total		705	100

Note: The highest number of dengue patients in a district is bolded.

**Table 2:** Descriptive analysis of continuous demographic and clinical data details for dengue patients in Wahidin hospital.

Variables	Min	Q1	Median	Mean	Q3	Max
LOS (days)	1.00	3.00	4.00	4.28	5.00	16
Age (years)	0.23	9.34	18.28	20.50	26.06	79
WBC (10^3 μL)	0.60	2.80	4.10	4.96	6.10	42.80
RBC (10^6 μL)	1.96	4.35	4.77	4.78	5.18	8.06
HGB (gr/dl)	5.70	12.00	13.20	13.24	14.60	22.20
HCT (%)	18	36	40	39.61	43	61
PLT (10^3 μL)	4	47	90	102	138	361

gr/dl: Grams per decilitre; 1 μL: 1 cell per microliter = 1 cell per cubic millimetre (mm^3^).

**Table 3:** Posterior hazard ratios for key parameters of the Weibull and Cox models.

Parameters	ICAR		Leroux		Independent	
	Mean	95% CI	Mean	95% CI	Mean	95% CI
**Parametric Weibull model**						
intercept	0.02	0.01; 0.02	0.02	0.01; 0.03	0.02	0.01; 0.02
Age	**0.72**	**0.66; 0.79**	**0.72**	**0.65; 0.78**	**0.71**	**0.65; 0.78**
Sex	1.02	0.95; 1.11	1.02	0.95; 1.11	1.02	0.94; 1.10
WBC	1.03	0.96; 1.11	1.03	0.96; 1.11	1.04	0.96; 1.11
RBC	**1.33**	**1.14; 1.55**	**1.31**	**1.12; 1.51**	**1.27**	**1.07; 1.50**
HGB	1.21	0.91; 1.62	1.19	0.90; 1.55	1.21	0.89; 1.63
HCT	0.80	0.58; 1.10	0.82	0.61; 1.11	0.83	0.57; 1.19
PLT	**0.87**	**0.81; 0.94**	**0.87**	**0.80; 0.94**	**0.87**	**0.80; 0.94**
*b*	13.13	11.30; 15.27	12.85	11.06; 14.61	13.14	11.53; 15.06
ρ	**-**	**-**	1.85	1.08; 2.67	**-**	**-**
$\sigma^{2}$	25.68	3.16; 880.09	1.13	1.003; 1.62	1.04	1.001; 1.17
**Semiparametric Cox model**						
Age	**0.82**	**0.75; 0.89**	**0.82**	**0.75; 0.89**	**0.82**	**0.75; 0.89**
Sex	1.01	0.94; 1.09	1.00	0.93; 1.09	1.01	0.93; 1.09
WBC	1.03	0.96; 1.1	1.03	0.96; 1.10	1.03	0.95; 1.10
RBC	**1.22**	**1.04; 1.44**	**1.21**	**1.02; 1.42**	**1.21**	**1.03; 1.42**
HGB	1.14	0.86; 1.5	1.12	0.82; 1.53	1.12	0.83; 1.53
HCT	0.79	0.56; 1.09	0.80	0.55; 1.16	0.80	0.56; 1.15
PLT	**0.89**	**0.82; 0.97**	**0.89**	**0.82; 0.96**	**0.89**	**0.82; 0.96**
ρ	-	-	1.82	1.05; 2.67	-	-
$\sigma^{2}$	48.39	3.99; 2683.83	1.04	1.0003; 1.21	2.70	1.03; 37.38

Bolded estimates indicate that the 95% credible interval does not include one.

**Table 4:** Estimation of spatial random effect (spatial frailty) hazard ratios for Weibull and Cox models. District numbers correspond to Table 1.

District	ICAR		Leroux		Independent	
	Mean	95% CI	Mean	95% CI	Mean	95% CI
Parametric Weibull model						
1	1.14	0.94; 1.39	1.12	0.80; 1.58	1.06	0.96; 1.30
2	1.20	0.80; 1.85	1.10	0.72; 1.81	1.03	0.88; 1.34
3	0.67	0.45; 0.95	0.77	0.46; 1.12	0.93	0.65; 1.05
4	0.93	0.63; 1.34	0.97	0.62; 1.46	0.99	0.79; 1.15
5	0.81	0.64; 1.02	0.87	0.58; 1.20	0.94	0.74; 1.05
6	1.04	0.72; 1.51	1.01	0.67; 1.51	1.01	0.85; 1.27
7	0.98	0.75; 1.26	1.00	0.70; 1.43	1.00	0.85; 1.17
8	0.96	0.78; 1.16	0.97	0.69; 1.35	1.00	0.86; 1.13
9	1.19	0.81; 1.71	1.10	0.75; 1.71	1.03	0.88; 1.33
10	0.96	0.81; 1.13	0.98	0.70; 1.35	0.99	0.86; 1.11
11	1.04	0.72; 1.46	1.04	0.69; 1.58	1.01	0.84; 1.24
12	0.96	0.61; 1.45	1.00	0.63; 1.54	0.99	0.80; 1.19
13	1.14	0.73; 1.86	1.07	0.68; 1.75	1.02	0.85; 1.34
14	1.15	0.51; 2.48	1.06	0.56; 2.08	1.01	0.83; 1.29
Semiparametric Cox model						
1	1.08	0.89; 1.31	1.04	0.85; 1.34	1.05	0.90; 1.26
2	1.10	0.76; 1.63	1.03	0.79; 1.39	1.03	0.82; 1.38
3	0.82	0.56; 1.16	0.94	0.66; 1.20	0.94	0.71; 1.15
4	0.93	0.63; 1.32	0.98	0.72; 1.30	0.97	0.75; 1.21
5	0.94	0.75; 1.16	0.98	0.77; 1.23	0.97	0.79; 1.14
6	1.01	0.71; 1.42	1.00	0.77; 1.33	1.00	0.80; 1.26
7	1.07	0.84; 1.37	1.04	0.81; 1.36	1.04	0.88; 1.28
8	0.98	0.80; 1.18	1.00	0.80; 1.25	0.99	0.84; 1.16
9	1.20	0.84; 1.73	1.05	0.82;1.48	1.06	0.86; 1.44
10	0.93	0.79; 1.10	0.97	0.77; 1.18	0.95	0.80; 1.09
11	0.98	0.70; 1.35	1.01	0.77; 1.34	0.99	0.79; 1.24
12	0.90	0.58; 1.33	0.98	0.72; 1.29	0.97	0.75; 1.20
13	1.07	0.70; 1.65	1.02	0.78; 1.39	1.02	0.80; 1.37
14	1.07	0.50; 2.20	1.02	0.70; 1.58	1.01	0.78; 1.35

**Table 5:** Goodness of fit of Weibull and Cox models.

PRIORS	Weibull		Cox	
	DIC	WAIC	DIC	WAIC
ICAR	2763.2	2823.96	2976.4	2967.77
Leroux	2752.6	**2812.76**	2963.8	2957.88
Independent	2752.5	2814.24	2963.5	2957.43

DIC: Deviance information criteria; WAIC: Watanabe–Akaike information criteria.

## Supplementary Materials

The following are available online at https://www.mdpi.com/1660-4601/17/3/878/s1, Table S1: The proportional hazard assumption test using Schoenfeld residuals, Code S1: R code for Bayesian Weibull spatial survival model using Leroux CAR prior, Code S2: R code for Bayesian Cox spatial survival model using Leroux CAR prior.
