# Supplementary material for: Bayesian Spatial Survival Models for Hospitalisation of Dengue: A Case Study of Wahidin Hospital in Makassar, Indonesia
Source: Int J Environ Res Public Health. 2020 Jan 30;17(3):878. doi: 10.3390/ijerph17030878 (PMC7037865; doi:10.3390/ijerph17030878)
Supplement: Supplementary file 1 [file ijerph-17-00878-s001.pdf]

Supplementary Materials

Table S1: The proportional hazard assumption test using Schoenfeld residuals

**Covariates**	**Global test**	**p**
**Age**	**0.11**	**0.11**
**Sex**	**0.63**	**0.63**
WBC	0.017	0.017
RBC	0.001	0.001
HGB	6.9e-07	6.9e-07
HCT	9.2e-07	9.2e-07
**PLT**	**0.71**	**0.71**
Age<br>Sex	0.11<br>0.42	0.22
**Age**<br>**PLT**	**0.17**<br>**0.84**	**0.39**
**Age**<br>**Sex**<br>**PLT**	**0.18**<br>**0.46**<br>**0.84**	**0.52**
Age<br>RBC<br>PLT	0.12862<br>0.00072<br>0.67132	0.00281
Age<br>Sex<br>PLT<br>RBC	0.13<br>0.52<br>0.67<br>0.00073	0.00711
WBC<br>RBC<br>HGB<br>HCT<br>PLT	0.04<br>0.00017<br>6.2e-06<br>4.6e-06<br>0.93645	4.1e-05

Code S1: R Code for Bayesian Weibull spatial survival model using Leroux CAR Prior

```
# Load R libraries
library(survival)
library (spdep)
library(ggplot2)
library(rgdal)
library(R2WinBUGS)
library(reshape2)
library(gridExtra)
library(SurvRegCensCov)

# Read in hospital data
Hospital_surv <- read.csv("Hospital_Wahidin.csv")

# Load shapefile
map <- readOGR("Kecamatan.shp")

# Get list of adjacency data
adjacent.data <- nb2WB(poly2nb(map))

# Clean data
Hospital_surv <- Hospital_surv[-which(is.na(Hospital_surv$WBC1)),]
Hospital_surv <- Hospital_surv[-which(is.na(Hospital_surv$HCT1)),]
Hospital_surv <- Hospital_surv[-which(is.na(Hospital_surv$PLT1)),]

# Duplicate LOS values before truncating for censored data
Hospital_surv$LOS_all <- Hospital_surv$LOS

#########################################################################
#the survival distribution is a truncated Weibull
#patients who are censored are given a missing value
# whilst patients who recover are given a zero in the censoring time vector t.cen
#########################################################################

Hospital_surv$t.cen1=1-Hospital_surv$t.cen
tcen2<- which(Hospital_surv$t.cen==0)
Hospital_surv$t.cen1[tcen2]=Hospital_surv$LOS[tcen2]
Hospital_surv$LOS[tcen2]=NA

#Standardised covariates
Hospital_surv$Sex_std <- (Hospital_surv$Sex-mean(Hospital_surv$Sex))/ sd(Hospital_surv$Sex)
Hospital_surv$Age_std <- (Hospital_surv$Age-mean(Hospital_surv$Age))/
sd(Hospital_surv$Age)
Hospital_surv$WBC1_std <- (Hospital_surv$WBC1-mean(Hospital_surv$WBC1))/
sd(Hospital_surv$WBC1)
Hospital_surv$RBC1_std <- (Hospital_surv$RBC1-mean(Hospital_surv$RBC1))/
sd(Hospital_surv$RBC1)
```

```
Hospital_surv$HGB1_std <- (Hospital_surv$HGB1-mean(Hospital_surv$HGB1))/
sd(Hospital_surv$HGB1)
Hospital_surv$HCT1_std <- (Hospital_surv$HCT1-mean(Hospital_surv$HCT1))/
sd(Hospital_surv$HCT1)
Hospital_surv$PLT1_std <- (Hospital_surv$PLT1-mean(Hospital_surv$PLT1))/
sd(Hospital_surv$PLT1)


#Define aliases
Sex <- Hospital_surv$Sex_std
Age <- Hospital_surv$Age_std
WBC1 <- Hospital_surv$WBC1_std
RBC1 <- Hospital_surv$RBC1_std
HGB1 <- Hospital_surv$HGB1_std
HCT1 <- Hospital_surv$HCT1_std
PLT1 <- Hospital_surv$PLT1_std
N <- nrow(Hospital_surv)
tcen1 <- Hospital_surv$t.cen1

# Create a dummy function containing model to be passed to WinBUGS
Model <-function(){
 for(j in 1:N.records){
  t[j] ~ dweib(b, lambda[j])%_%I(t.cen1[j],)
  c[j] ~ dweib(b, lambda[j])%_%I(t.cen1[j],)
 }

 # Leroux prior for spatial random effects
 for(i in 1:N){    S[i] ~ dnorm(mean.S[i], prec.S[i])
  A[i] <- (rho * num[i] + 1 - rho)
  prec.S[i] <- A[i] / sigma.S2
  mean.S[i] <- rho * sum(W.S[cum[i] + 1:cum[i+1]]) / A[i]}
 for(h in 1:sumnum){
  W.S[h] <- S[adj[h]]}
 for (j in 1:N.records){
  log(lambda[j])<-(beta[1]+beta[2]*Age[j]+
beta[3]*Sex[j]+beta[4]*WBC1[j]+beta[5]*RBC1[j]+beta[6]*HGB1[j]+beta[7]*HCT1[j]+
beta[8]*PLT1[j]+S[Area[j]])
    }

 for (k in 1:8) {
  beta[k] ~ dnorm(0, 0.01)
 }
 b ~ dgamma(2, 0.5)
 rho ~ dunif(0, 1)
 sigma.S2 ~ dgamma(1, 0.1)
}

# Save model as .bug file in temporary location
fp.model <- file.path(tempdir(), "Model_Bayesian_spatial_survival.bug")
write.model(Model, fp.model)
```

```
# Fixed values as a named list
data <- c(
  list(
    N = 14,
    N.records = nrow(Hospital_surv),
    Area = Hospital_surv$District,
    t = Hospital_surv$LOS,
    t.cen1 =Hospital_surv$t.cen1,
    adj = adjacent.data$adj,
    num = adjacent.data$num,
    cum = c(cumsum(adjacent.data$num) - adjacent.data$num, sum(adjacent.data$num)),
    sumnum = sum(adjacent.data$num),
    Sex = Sex,
    Age = Age,
    WBC1 =WBC1,
    RBC1 = RBC1,
    HGB1= HGB1,
    HCT1= HCT1,
    PLT1= PLT1
  )
)

# Initial values for stochastic parameters
inits <- function() {list(
  b = 0.5,
  S = rnorm(14, 0, 0.1),
  beta = rep(0, 8),
  rho= 0.6,
  sigma.S2 = 0.1
)}

# MCMC parameters
M.burnin <- 5000   # Number of burn-in iterations (discarded)
M <- 5000         # Number of iterations retained
n.thin <- 1        # Thinning factor

# Fit the model using WinBUGS
set.seed(1)
MCMC <- bugs(
  data = data,
  inits = inits,
  parameters.to.save = c("b", "S", "beta", "rho", "sigma.S2", "lambda", "c"),
  model.file = fp.model,
  n.chains = 1,
  n.burnin = M.burnin,
  n.iter = M.burnin + (M * n.thin),  # Total iterations
  n.thin = n.thin,
  DIC = TRUE
  )
```

Code S2: R Code for Bayesian Cox spatial survival model using Leroux CAR Prior

```
# Load R libraries
library(survival) # for coxph(), Surv()
library (spdep) # for poly2nb(), nb2mat, nb2WB()
library(ggplot2)
library(rgdal) # for readOGR
library(R2WinBUGS) #For bugs(), write.model()
library(reshape2)
library(gridExtra) # For grid.arrange()

# Load hospital data
Hospital_surv <- read.csv("Hospital_Wahidin_Cox.csv")

# Load shapefile
map <- readOGR("Kecamatan.shp")

# Get list of adjacency data
adjacent.data <- nb2WB(poly2nb(map))

# Clean data
Hospital_surv <- Hospital_surv[-which(is.na(Hospital_surv$WBC1)),]
Hospital_surv <- Hospital_surv[-which(is.na(Hospital_surv$HCT1)),]
Hospital_surv <- Hospital_surv[-which(is.na(Hospital_surv$PLT1)),]

#Standardised covariates
Hospital_surv$Sex_std <- (Hospital_surv$Sex-mean(Hospital_surv$Sex))/ sd(Hospital_surv$Sex)
Hospital_surv$Age_std <- (Hospital_surv$Age-mean(Hospital_surv$Age))/
sd(Hospital_surv$Age)
Hospital_surv$WBC1_std <- (Hospital_surv$WBC1-mean(Hospital_surv$WBC1))/
sd(Hospital_surv$WBC1)
Hospital_surv$RBC1_std <- (Hospital_surv$RBC1-mean(Hospital_surv$RBC1))/
sd(Hospital_surv$RBC1)
Hospital_surv$HGB1_std <- (Hospital_surv$HGB1-mean(Hospital_surv$HGB1))/
sd(Hospital_surv$HGB1)
Hospital_surv$HCT1_std <- (Hospital_surv$HCT1-mean(Hospital_surv$HCT1))/
sd(Hospital_surv$HCT1)
Hospital_surv$PLT1_std <- (Hospital_surv$PLT1-mean(Hospital_surv$PLT1))/
sd(Hospital_surv$PLT1)

# Define aliases
Sex <- Hospital_surv$Sex_std
Age <- Hospital_surv$Age_std
WBC1 <- Hospital_surv$WBC1_std
RBC1 <- Hospital_surv$RBC1_std
HGB1 <- Hospital_surv$HGB1_std
HCT1 <- Hospital_surv$HCT1_std
PLT1 <- Hospital_surv$PLT1_std
N <- nrow(Hospital_surv)                    #Number of subjects (patients), N=705
K <- length(unique(Hospital_surv$District))   #Number of Districts; K =14
```

```
obs.t <- Hospital_surv$LOS
tcen  <- Hospital_surv$tcen
tmp <- obs.t[tcen > 0] %>% unique
tmp <- tmp[order(tmp)]
time<-c(tmp, max(obs.t))                    #time vector (unique observed LOS)
T <- length(time) - 1                 #time interval; T=13


# Create Y and dN
eps=0.000001
Y <- matrix(NA, N, T)
dN <- matrix(NA, N, T)
for(i in 1:N) {
 for(j in 1:T) {
  Y[i, j] <- ifelse(obs.t[i] - time[j] + eps >= 0, 1, 0)
  dN[i, j] <- Y[i, j] * ifelse(time[j + 1] - obs.t[i] - eps >= 0, 1, 0) * tcen[i]
 }
}

# Create a dummy function containing model to be passed to WinBUGS
Model <-function() {
 for(j in 1:T) {
  for(i in 1:N) {
   dN[i,j] ~ dpois(Idt[i, j]) # Likelihood
   Idt[i,j]<-Y[i,j]*exp(beta[1]*Age[i]+
beta[2]*Sex[i]+beta[3]*WBC1[i]+beta[4]*RBC1[i]+beta[5]*HGB1[i]+beta[6]*HCT1[i]+
beta[7]*PLT1[i]+ S[Area[i]]) * dL0[j] # Intensity
  }
  dL0[j] ~ dgamma(mu[j], c)
  mu[j] <- dL0.star[j] * c # prior mean hazard
   dL0.star[j] <- r * (time[j + 1] - time[j])
 }
#Prior for covariates
 for (k in 1:7) {
  beta[k] ~ dnorm(0, 0.01)
 }

 # Leroux prior for spatial random effects
 for(k in 1:K){
  S[k] ~ dnorm(mean.S[k], prec.S[k])
  A[k] <- (rho * num[k] + 1 - rho)
  prec.S[k] <- A[k] / sigma.S2
  mean.S[k] <- rho * sum(W.S[cum[k] + 1:cum[k+1]]) / A[k]}
 for(h in 1:sumnum){
  W.S[h] <- S[adj[h]]}

 rho ~ dunif(0, 1)
 sigma.S2 ~ dgamma(1, 0.1)
}

# Save model as .bug file in temporary location
```

```
fp.model <- file.path(tempdir(), "Model_Cox_spatial_survival.bug")
write.model(Model, fp.model)

# Fixed values as a named list
data <- c(list(
   N = N,
   T = T,
   K = K,
   Age=Age,
   Sex =Sex,
   WBC1 = WBC1,
   RBC1 = RBC1,
   HGB1= HGB1,
   HCT1=HCT1,
   PLT1= PLT1,
   Area = Hospital_surv$District,
   adj = adjacent.data$adj,
   num = adjacent.data$num,
   cum = c(cumsum(adjacent.data$num) - adjacent.data$num, sum(adjacent.data$num)),
   sumnum = sum(adjacent.data$num),
   time=time,
   dN=dN,
   Y=Y,
   c = 0.001,
   r = 0.1
  ))

# Initial values for stochastic parameters
inits <- function() {list(
  beta = rep (0, 7),
  dL0=rep(1,T),
  S = rnorm(K, 0, 0.1),
  rho= 0.6,
  sigma.S2 = 0.1
)}

# MCMC parameters
M.burnin <- 5000  # Number of burn-in iterations (discarded)
M <- 5000            # Number of iterations retained
n.thin <- 1            # Thinning factor

# Fit the model using WinBUGS
set.seed(1)
MCMC <- bugs(
  data = data,
  inits = inits,
  parameters.to.save = c("dL0", "rho",  "sigma.S2", "S", "beta"),
  model.file = fp.model,
  n.chains = 1,
  n.burnin = M.burnin,
  n.iter = M.burnin + (M * n.thin), # Total iterations
```

```
  n.thin = n.thin,
  DIC = TRUE
)
```
